# Hypertriglyceridemia-Induced Pancreatitis as a Result of Quetiapine use

**DOI:** 10.7759/cureus.12074

**Published:** 2020-12-14

**Authors:** Eric Landa, Erika Vigandt, Ismail Ganim, Joseph Abraham, Talhah Siraj

**Affiliations:** 1 Internal Medicine, Unity Health-White County Medical Center, Searcy, USA; 2 Internal Medicine, Brooklyn Hospital, Brooklyn, USA

**Keywords:** pancreas, pancreatitis, quetiapine, gastrointestinal, drug induced pancreatitis

## Abstract

Pancreatitis is caused by a number of different etiologies, most commonly caused by gallstone induced, alcohol, and familial hypertriglyceridemia. Other less common causes include trauma, medications, and autoimmune conditions. Drug-induced pancreatitis (DIP) is only responsible for less than 2% of all cases but is a very important etiology that has been observed with increasing frequency in the acute setting. Here we present a case of recurrent pancreatitis with no other risk factors except for the initiation of quetiapine approximately six months prior to the first episode.

## Introduction

Associated with over 100,000 hospitalizations per year, pancreatitis remains the number one gastrointestinal reason for hospitalization in the United States [1]. The healthcare and financial expenditure remains high, not just in the United States but worldwide. The most common causes include gallstones and alcohol, which compromise up to 95% of all cases. Hypertriglyceridemia, trauma, endoscopic retrograde cholangiopancreatography (ERCP), and medications compromise the remaining [2]. With the World Health Organization listing over 500 medications associated with acute pancreatitis [3], the recognition of drug-induced pancreatitis (DIP) is very important in stopping the recurrence and possible complications that can be seen with multiple/continuous bouts of pancreatitis such as abscess formation, necrotic transformation, etc. Herein, we present a case of drug-induced recurrent pancreatitis. 

## Case presentation

**Figure 1:**
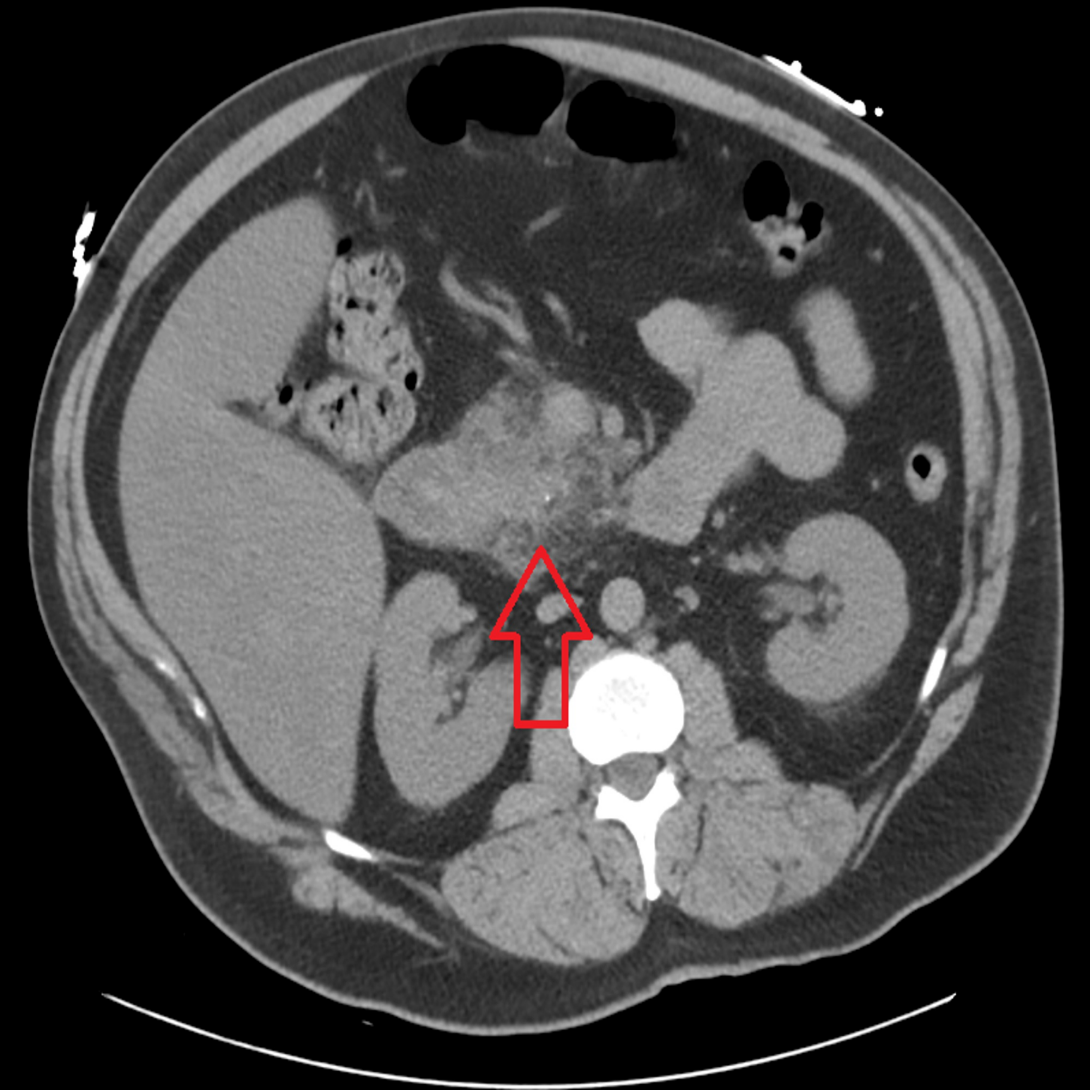
CT scan showing an inflammatory change located within the pancreatic head consistent with pancreatitis.

**Figure 2:**
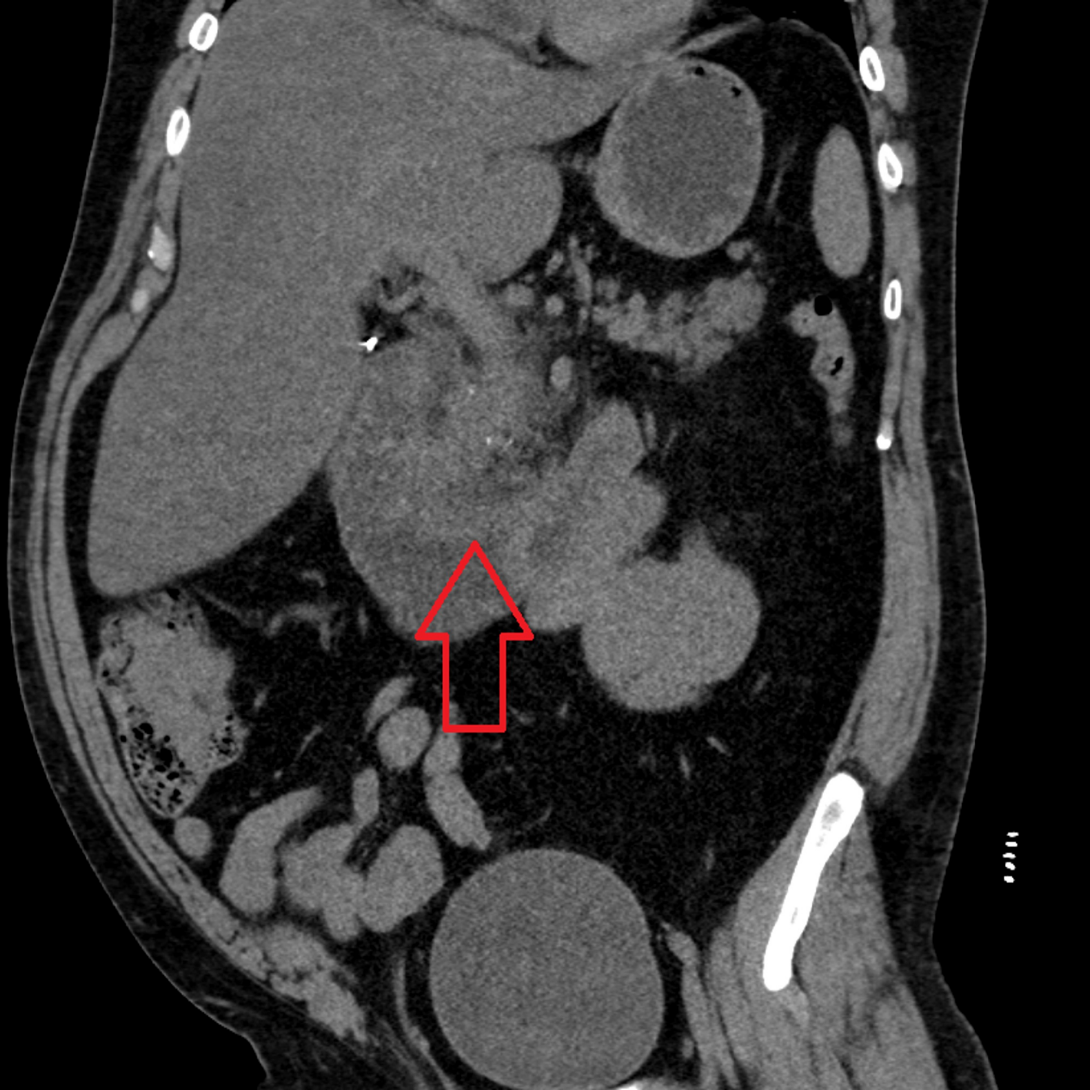
CT scan showing pancreatic fluid collection.

A 46-year-old Caucasian male with past medical history of noninsulin dependent Type II diabetes, hypertension, recurrent pancreatitis episodes, asthma, and gout presented to the ED after experiencing severe right upper quadrant pain radiating to his back that started today. The patient stated that he was standing on the side of the road with his motorcycle because he was fixing a certain part that had become loose when suddenly he started to experience severe right upper quadrant pain that made him pass out. He denies hitting his head and stated he was out for approximately two hours (he looked at his watch and noticed how much time had passed), when he awoke he went straight to the hospital. He described the pain as 10/10, burning in nature, associated with an episode of nonbloody, nonbilious vomiting. He denied having any diarrhea, chest pain, shortness of breath, or trauma of any kind. Upon admission, his vitals were within normal limits, blood glucose: 565 mg/dL, calcium: 11 mg/dL, aspartate aminotransferase (AST): 52 u/L, alanine transaminase (ALT): 64 u/L, bicarbonate: 12 mmol/L, anion gap: 18 mmol/L, B-hydroxybutyrate: 0.82 mmol/L, triglyceride: 1213 mg/dL, lipase: 374 u/L, urinalysis was significant for protein 4+, ketones 2+. A CT of the abdomen/pelvis without contrast was significant for “acute edematous pancreatitis centered on the pancreatic head and uncinate process along with pancreatic fluid collection (Figures 1-2).” He was given lactated ringer's solution at 150 mL/h, kept nothing by mouth (NPO) initially, and admitted to the ICU for further management due to also having diabetic ketoacidosis (DKA). He was initiated on an insulin drip w/dextrose, fenofibrate, and atorvastatin. He has a history of five previous episodes of pancreatitis with a cholecystectomy performed during his second episode. There is no family history of pancreatitis or familial hypertriglyceridemia. Social history is significant for smoking two packs/day x 15 years, quit two years ago, denies any alcohol and/or drug use. A review of his medications showed that he had started taking quetiapine 150 mg once a day for depression about six months prior to his first episode. His DKA resolved after two days, his quetiapine was discontinued at this time. A repeat CT scan revealed improvement of the inflammation with no signs of necrotic changes or abscess formation. Once he regained his appetite back, he was started on a liquid diet which was then progressed to regular diet. He was discharged home straight from the ICU two days later and was counseled on following up with his primary care physician to review his medications.

## Discussion

**Figure 3:**
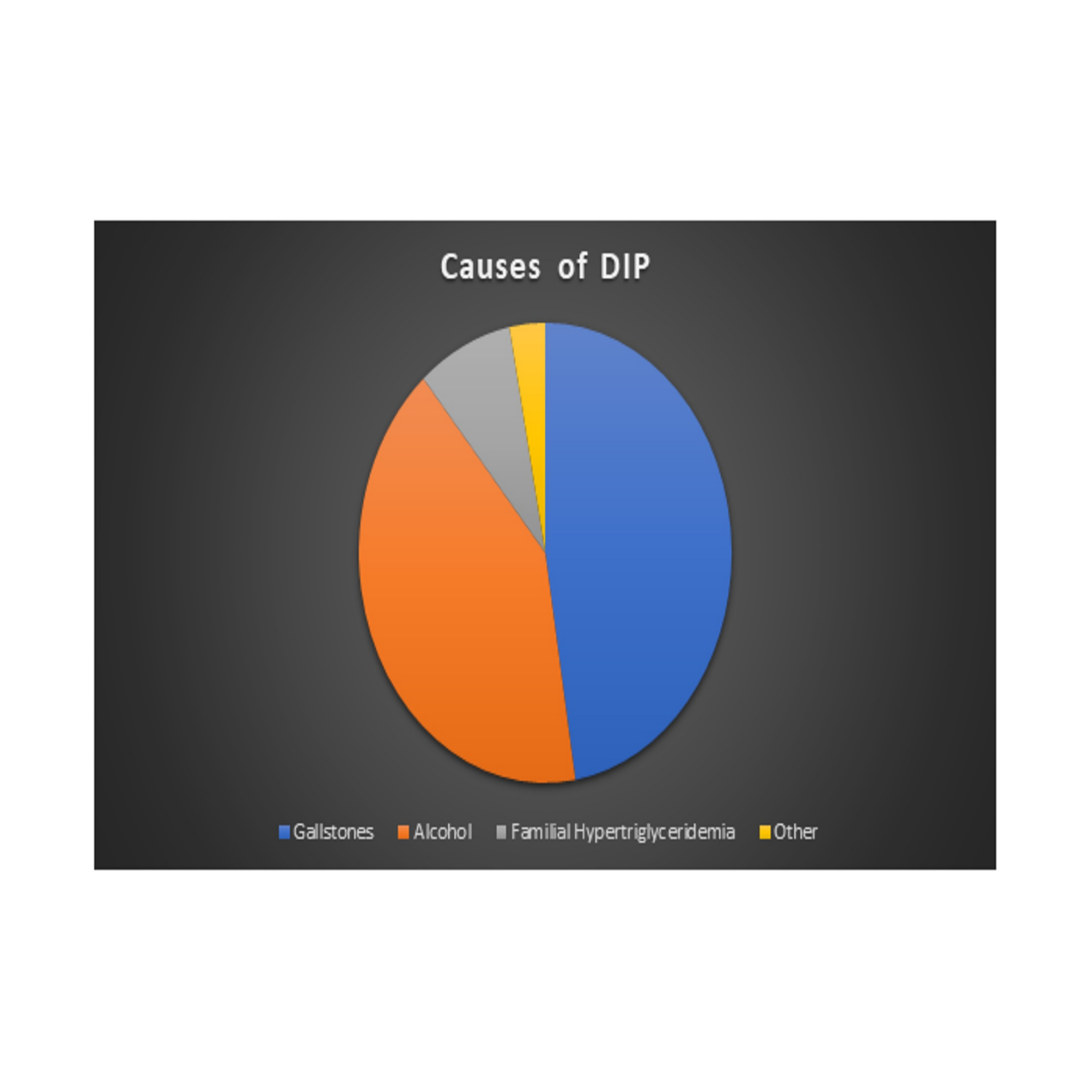
Most common causes of pancreatitis. DIP, drug-induced pancreatitis

Although very uncommon, the prevalence of DIP seems to be growing as more cases are being reported [4-5]. The exact mechanism of injury is not known, although it likely varies depending on the class of drugs involved. The mechanisms proposed include pancreatic/biliary duct constriction, cytotoxic effects, metabolic effects, accumulation of a toxic metabolite or intermediary, idiosyncratic, and/or hypersensitivity reaction [6-7]. The number of acute pancreatitis cases caused by drugs is small (0.3%-1.4%) [8] that it is often hard to make such a diagnosis because of comorbidities or alcohol intake which might cause a physician to not consider the medications as a possible culprit (Figure 3). Liou et al. proposed a classification system for the different drugs that have been implicated in pancreatitis [9]. It consists of four categories (I-IV), with the first one expanded to Ia and Ib, on the basis of the number of case reports, available rechallenge data (a rechallenge test is when a drug thought to be the culprit is restarted on the same patient to see whether it will result in an episode of pancreatitis), consistent latency period, and ability to exclude other causes of acute pancreatitis. Latency is defined as the time elapsed between exposure to a drug and first clinical manifestation of signs and symptoms of acute pancreatitis. In the opinion of Badalov et al. consistent latency among the available case reports, despite the lack of a rechallenge, may strongly suggest such etiology of acute pancreatitis [10-11]. 

Quetiapine-induced hypertriglyceridemia causing acute pancreatitis has been reported in only six published case reports [11]. The most effective way to diagnose DIP is through a rechallenge test which is when the offending agent is stopped and resolution of pancreatitis is established and reintroducing the drug causes acute pancreatitis. Given the fact that our patient developed his first episode six months after starting quetiapine and lacking any other possible risk we strongly believe quetiapine to be the culprit. Nevertheless, it cannot be fully proven without performing the rechallenge test and has not been done in this case due to ethical concerns. 

## Conclusions

We have seen many different etiologies that can result in pancreatitis and though not as common, medications should never be overlooked. If no obvious cause is identified, it is wise to always think of any new medications a patient might have recently started taking. We present this case in the hopes that it will help make physicians and providers aware of DIP and lead to early discontinuation of the offending agent.

## References

[1] Peery AF, Crockett SD, Murphy CC Burden and cost of gastrointestinal, liver, and pancreatic diseases in the United States: update 2018. Gastroenterology.

[2] Yang AL, Vadhavkar S, Singh G (2008). Epidemiology of alcohol-related liver and pancreatic disease in the United States. Arch Intern Med.

[3] Nitsche CJ, Jamieson N, Lercha MM, Mayerle JV (2010). Drug induced pancreatitis. Best Pract Res Clin Gastroenterol.

[4] Rünzi M, Layer P (1996). Drug-associated pancreatitis: facts and fiction. Pancreas.

[5] Spanier MBW, Tuynman HARE, van der Hulst RWM, Dijkgraaf MGW, Bruno MJ (2011). Acute pancreatitis and concomitant use of pancreatitis-associated drugs. Am J Gastroenterol.

[6] Jones MR, Hall OM, Kaye AM, Kaye AD (2015). Drug-induced acute pancreatitis: a review. Ochsner J.

[7] Underwood TW, Frye CB (1993). Drug-induced pancreatitis. Clin Pharm.

[8] Lankisch PG, Dröge M, Gottesleben F (1995). Drug induced acute pancreatitis: incidence and severity. Gut.

[9] Liou LS, Hung YJ, Hsieh CH, Hsiao FC (2014). Aggravation of hypertriglyceridemia and acute pancreatitis in a bipolar patient treated with quetiapine. Yonsei Med J.

[10] Badalov N, Baradarian R, Iswara K, Li J, Steinberg W, Tenner S (2007). Drug-induced acute pancreatitis: an evidence-based review. Clin Gastroenterol Hepatol.

[11] Ksiądzyna D (2011). Drug-induced acute pancreatitis related to medications commonly used in gastroenterology. Eur J Intern Med.

